# A Framework for Final Drive Simultaneous Failure Diagnosis Based on Fuzzy Entropy and Sparse Bayesian Extreme Learning Machine

**DOI:** 10.1155/2015/427965

**Published:** 2015-02-05

**Authors:** Qing Ye, Hao Pan, Changhua Liu

**Affiliations:** ^1^School of Computer Science and Technology, Wuhan University of Technology, Wuhan 430000, China; ^2^Yangtze University College of Technology and Engineering, Jingzhou 430023, China

## Abstract

This research proposes a novel framework of final drive simultaneous failure diagnosis containing feature extraction, training paired diagnostic models, generating decision threshold, and recognizing simultaneous failure modes. In feature extraction module, adopt wavelet package transform and fuzzy entropy to reduce noise interference and extract representative features of failure mode. Use single failure sample to construct probability classifiers based on paired sparse Bayesian extreme learning machine which is trained only by single failure modes and have high generalization and sparsity of sparse Bayesian learning approach. To generate optimal decision threshold which can convert probability output obtained from classifiers into final simultaneous failure modes, this research proposes using samples containing both single and simultaneous failure modes and Grid search method which is superior to traditional techniques in global optimization. Compared with other frequently used diagnostic approaches based on support vector machine and probability neural networks, experiment results based on *F*_1_-measure value verify that the diagnostic accuracy and efficiency of the proposed framework which are crucial for simultaneous failure diagnosis are superior to the existing approach.

## 1. Introduction

With sustained increase of work condition complexity, simultaneous failures occur more frequently in final drive which is the pivotal part of car and seriously affect running status and comfort and safety of car. Final drive is mainly consisting of a pair of gears which are meshing together when car runs. Owing to the complex structure, a certain function disorder in final drive usually stems from more than one single failure at the same time which is called simultaneous failure. Traditional manual technology cannot accomplish simultaneous failure diagnosis (SFD). This paper is focusing on final drive simultaneous failure diagnosis which is essential for auto manufacturer and maintenance industry.

Failure diagnosis by using vibration signal is almost the most frequently used approach because vibration signal is relatively precise and accurate against diagnosis based on sound. It can be divided into three main steps: feature extraction, training diagnostic models, and failure mode identification. The vibration signal collected from final drive has the characteristics of being nonlinear and nonstationary, and it is enclosed with a lot of uncorrelated and superfluous information. It is impossible to extract valid failure mode information from original vibration signal because of the noise and interference embedded in it. The frequently used preprocessed methods include wavelet analysis [1, 2], wavelet package transform (WPT) [3, 4], and empirical mode decomposition (EMD) [5]. Wavelet package transform is suitable for nonstationary vibration signal by decomposing original signal into several subfrequency bands which contains different failure information and effectively reduces noise interference.

Data contained in preprocessed signal is high-dimensional so that it cannot be directly inputted into diagnostic system. Feature extraction has a deep effect on accuracy and reliability of failure diagnosis. Recently, researchers have introduced entropy into the field of feature extraction including approximate entropy, sample entropy [6], and fuzzy entropy [7]. Compared with approximation entropy and sample entropy which are based on Heaviside step function which is mutational at the classification boundary, fuzzy entropy eliminates the influence of baseline drift of data and guarantees the entropy to vary smoothly and continuously with similarity tolerance [8] so that it is excellent in measuring complexity and self-similarity of the preprocessed vibration signal and fully reflecting changes of the vibration performance of mechanical equipment [9].

In recent years, many machine learning methods are applied in failure diagnosis including support vector machine (SVM) [10], artificial neural networks (ANN) [11], extreme learning machine (ELM) [12, 13], and kernel extreme learning machine (KELM) [14]. ELM is single-hidden-layer feedforward neural networks and without human intervention in tuning parameters which differs from SVM and ANN and makes it superior in high generalization and less learning time. KELM apply kernel function to ELM to improve generalization and nonlinear approximation ability [15]. However, computational cost and memory cost of KELM are high with regard to large scale problem. Recently, Bayesian methods are employed into ELM to learn the output weights by estimating the probability distribution of output with high generalization. Soria-Olivas and Gómez-Sanchis proposed Bayesian extreme learning machine [16] for linear regression without solving classification problem. Sparse Bayesian extreme learning machine (SBELM) [17] is a novel method for finding the sparse representatives of hidden layer output weights by imposing a hyperparameter on each weight. During learning phase, SBELM tunes some output weights into zero to obtain compact model. In summary, SBELM has the advantages of probability output, high generalization, sparsity, and fast training speed. To solve the problem of simultaneous failure diagnosis, a proper classifier has to offer the probability of all possible failures. In this research, the proposed framework constructs classifiers based on paired SBELM in which each classifier based on SBELM is trained by a pair of single failure samples. The paired SBELM effectively reflect the probability distribution of failure modes. In general, only single failure samples are used for constructing diagnostic models. Since it is impossible to collect all combinations of existing single failure modes for training, the proposed framework can effectively solve the practical bottleneck in simultaneous failure diagnosis. With the purpose of recognizing simultaneous failure modes, use both single and simultaneous failure samples and Gird search method to generate optimal decision threshold which could convert probability result of classifier into final multiple failure modes. Considering that partial matching is valid and instrumental in simultaneous failure diagnosis, this research adopts *F*_1_-measure to evaluate the performance of the proposed framework.

This paper is organized as follows: Section 2 presents the proposed framework.  Section 3 presents the experiment setup, data acquisition, and preprocessing. The results of experiment are discussed in Section 4. Finally, a conclusion is given in Section 5.

## 2. The Proposed Framework for Final Drive Simultaneous Failure Diagnosis

### 2.1. Feature Extraction Based on Wavelet Package Transform and Fuzzy Entropy

#### 2.1.1. Wavelet Package Transform

In failure diagnosis, one of the key points is extraction of features from original vibration signal which is nonstationary for mechanical equipment. Wavelet package transform (WPT) is an extended form of wavelet transform to analyse nonstationary and non-linear signal and to supply better partition of frequency band because the same frequency bandwidths can provide good resolution regardless of high and low frequencies [18]. As a multiresolution analysis method, WPT can effectively preprocess nonstationary vibration signal in both time domain and frequency domain. Two-scale equation of WPT is shown below, in which *h*_0*k*_ and *h*_1*k*_ represent the filter coefficients:

(1)  x2nt=2∑k∈Zh0kxn2t−k, x2n+1t=2∑k∈Zh1kxn2t−k.

The recursion formula of wavelet package coefficient is

(2) $$\begin{array}{c} d_{k}^{j+1,2n}=\underset{l}{\sum }h_{0\left(2l-k\right)}d_{l}^{j,n},\;\;d_{k}^{j+1,2n+1}=\underset{l}{\sum }h_{1\left(2l-k\right)}d_{l}^{j,n}. \end{array}$$

#### 2.1.2. Fuzzy Entropy

When failure occurred in final drive, the complexity of oscillation feature will change; hence we should extract representative features containing in the signal. Fuzzy entropy is an extension of Shannon entropy and fuzzy sets [19]. The procedure of fuzzy entropy is described as follows.

(1) Consider a time series with the length of *N*  {*x*(*i*) : 1 ≤ *i* ≤ *N*}. For given *m*, *n*, and *r*, construct a vector set in the form of {*X*_*i*_^*m*^, *i* = 1,2,…, *N* − *m* + 1} in which each vector contains *m* sequential elements starting from *x*(*i*) shown as

(3) Xim=xi,xi+1,…,xx+m−1−x0i,i=1,2,…,N−m+1,

where *x*_0_(*i*) is the average of vector *X*_*i*_^*m*^.

(2) Define the distance *d*_*ij*_^*m*^ between *X*_*i*_^*m*^ and *X*_*j*_^*m*^ where *i*, *j* = 1,2,…, *N* − *m*, *i* ≠ *j* as follows:

(4) $$\begin{array}{c} d_{ij}^{m}=\underset{k\in \left(0,m-1\right)}{\max}\left\{\left|\left[x\left(i+k\right)-x_{0}\left(i\right)\right]-\left[x\left(j+k\right)-x_{0}\left(j\right)\right]\right|\right\}. \end{array}$$

(3) Calculate similarity between *X*_*i*_^*m*^ and *X*_*j*_^*m*^ using fuzzy function:

(5) $$\begin{array}{c} D_{ij}^{m}=\mu \left(d_{ij}^{m},n,r\right)=e^{-\mathrm{In}2{\left(d_{ij}^{m}/r\right)}^{n}}. \end{array}$$

(4) Define function *ϕ* ^*m*^ as follows:

(6) $$\begin{array}{c} \phi^{m}\left(n,r\right)=\frac{1}{N-m}\overset{N-m}{\underset{i=1}{\sum }}\left(\frac{1}{N-m+1}\overset{N-m}{\underset{j=1,j\neq i}{\sum }}D_{ij}^{m}\right). \end{array}$$

(5) Change *m* to *m* + 1 and repeat step (1) to (4):

(7) $$\begin{array}{c} \phi^{m+1}\left(n,r\right)=\frac{1}{N-m}\overset{N-m}{\underset{i=1}{\sum }}\left(\frac{1}{N-m+1}\overset{N-m}{\underset{j=1,j\neq i}{\sum }}D_{ij}^{m+1}\right). \end{array}$$

(6) Fuzzy entropy of sequence {*x*(*i*) : 1 ≤ *i* ≤ *N*} is defined as follows:

(8) $$\begin{array}{c} \text{FuzzyEn}\left(m,n,r\right)=\underset{N\to \infty }{\lim}\left[\mathrm{In}\phi^{m}\left(n,r\right)-{\mathrm{In}\phi }^{m+1}\left(n,r\right)\right]. \end{array}$$

(7) If the length *N* is finite, FuzzyEn(*m*, *n*, *r*) can be changed as follows:

(9) $$\begin{array}{c} \text{FuzzyEn}\left(m,n,r\right)=\mathrm{In}\phi^{m}\left(n,r\right)-{\mathrm{In}\phi }^{m+1}\left(n,r\right). \end{array}$$

### 2.2. Sparse Bayesian Extreme Learning Machine (SBELM)

Given a preprocessed data set *D* = (*x*_*i*_, *t*_*i*_), *i* = 1,…, *N*, *x*_*i*_ ∈ *R* ^*n*^, *t*_*i*_ ∈ *R* ^*m*^. The output function of ELM with *L* hidden nodes is shown as follows:

(10) $$\begin{array}{c} f\left(x\right)=\overset{L}{\underset{i=1}{\sum }}\beta_{i}h_{i}\left(x\right)=\beta \cdot H\left(x\right), \end{array}$$

where *β* = [*β*_1_,…,*β*_*L*_]^*T*^ is output weight connecting hidden nodes and output nodes; *H*(*x*) = [*h*_1_(*x*),…, *h*_*L*_(*x*)] is the hidden layer output matrix for input *x* in which *h*_*i*_(*x*) is the hidden output of the *i*th hidden node. Equation (10) can be written as follows:

(11) $$\begin{array}{c} H\beta =T, \end{array}$$

where *T* is the training data target matrix. SBELM learns output weight by using Bayesian method instead of by calculating Moore-Penrose generalized inverse of *H* [17]. The hidden layer output *H* becomes the input of SBELM. Treat each training sample as an independent Bernoulli event so that probability *p*(*t*∣*x*) satisfies Bernoulli distribution. Apply sigmoid function to convert the predicted output *Y*(*h*; *β*) as follows:

(12) $$\begin{array}{c} \sigma \left(Y\left(h;\beta \right)\right)=\frac{1}{\left(1+\exp\left(-Y\left(h;\beta \right)\right)\right)}. \end{array}$$

The likelihood function of sample set is expressed as follows:

(13) $$\begin{array}{c} p\left(t\;|\;H,\beta \right)=\overset{N}{\underset{j=1}{\prod }}{\sigma \left(Y\left(h;\beta \right)\right)}^{t_{j}}{\left[1-\sigma \left(Y(h;\beta )\right)\right]}^{1-t_{j}}, \end{array}$$

where *t*_*j*_ is the target of training sample *x*_*j*_, *Y*(*h*; *β*) = *hβ*, and *t*_*j*_ ∈ {0,1}. Conditioned on a hyperparameter *α*_*j*_, zero-mean Gaussian prior distribution over *β*_*i*_ is as follows:

(14) $$\begin{array}{c} p\left(\beta \;|\;\alpha \right)=\overset{L}{\underset{i=1}{\prod }}\frac{\alpha_{i}}{\sqrt{2\pi }}\exp\left(-\frac{\alpha_{i}\beta_{i}^{2}}{2}\right). \end{array}$$

The typical step of SBELM is to establish the distribution of marginal likelihood over *t* conditioned on *α* and *H* and determine *α* by maximizing the marginal likelihood *p*(*t*∣*H*, *α*) by Laplace approximation method:

(15) argmax ln⁡⁡pt ∣ β,Hpβ ∣ α  =argmaxln⁡∏j=1Nyjtj1−yj1−tj       ∏j=1Nyjtj1−yj1−tj+ln⁡⁡∏i=1Lαi2πexp⁡⁡−αiβi22  =argmax∑j=1Ntjln⁡⁡yj+1−tjβTAβ2         ∑j=1N·ln⁡⁡1−yj−βTAβ2+const,

where *y*_*j*_ = *σ*(*Y*(*h*_*j*_; *β*)), *A* = diag⁡(*α*), and const = ∑_*i*=1_^*L*^ln⁡⁡*α*_*i*_ − 1/2ln⁡⁡2*π*. Then, make quadratic approximation for log of posterior probability:

(16) $$\begin{array}{c} \nabla \beta \nabla \beta \ln\left\{p\left(t\;|\;\beta ,H\right)p\left(\beta \;|\;\alpha \right)\right\}=-\left(H^{T}KH+A\right), \end{array}$$

where *K* is a diagonal matrix in which *k*_*j*_ = *y*_*j*_(1 − *y*_*j*_) with *j* = 1,…, *N*. Therefore, the center and covariance matrix of Gauss distribution of *β* expressed as *β*′ and Φ are obtained as follows:

(17) $$\begin{array}{c} \beta^{\prime }=\Phi H^{T}Kt^{\prime },\;\;\Phi ={\left(H^{T}KH+A\right)}^{-1}, \end{array}$$

where *t*′ = *Hβ* + *K* ^−1^(*t* − *y*). By obtaining Gauss approximation of *β*, the log of marginal likelihood is represented as follows:

(18) Lα=ln⁡⁡pt ∣ α,H=−12Nln⁡⁡2π+ln⁡⁡C+t′TC−1t′,

where *C* = *K* + *HAH* ^*T*^. By setting the differential of *L*(*α*) with respect to *α* as 0, update the hyperparameter *α* as follows:

(19) $$\begin{array}{c} \alpha_{i}=\frac{1-\alpha_{i}\Phi_{ii}}{{{(\beta }_{i^{\prime }})}^{2}}. \end{array}$$

The main procedure of SBELM is described as follows.

1. Initialize *α*_*i*_ and *β*_*i*_ randomly with *i* = 1,…, *L*.
2. By utilizing Laplace approximation approach, obtain approximated Gauss distribution of *β* and update *β*′ and Φ by using (17).
3. By maximizing the marginal likelihood, utilize (19) to update hyperparameter *α* until reaching the termination criteria.
4. By tuning some *β*_*i*_ into 0, obtain the sparse representation of hidden layer output weight.
5. For an unknown sample *x*_*u*_, utilize (12) to predict probability distribution *p*(*t*∣*x*_*u*_, *β*′).

### 2.3. Paired SBELM

**Figure 1:**
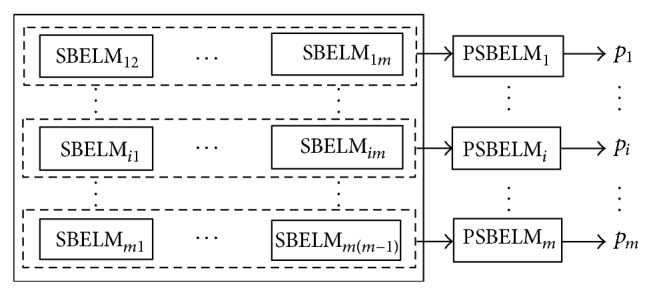
Structure of paired SBELM for simultaneous failure diagnosis.

SBELM is excellent in solving binary classification by obtaining probability distribution of each class *p*(*t*∣*x*, *β*′). With the purpose of final drive simultaneous failure diagnosis in which training samples of single failure are ample while training samples of simultaneous failure are scarce, this paper combines the state-of-the-art coupling approach proposed in [20] with SBELM to construct a set of paired SBELM (PSBELM) classifiers expressed as [PSBELM_1_,…, PSBELM_*m*_] for a *m*-label classification problem shown in Figure 1 and each paired classifier PSBELM_*i*_ = [SBELM_*i*1_,…, SBELM_*ij*_,…, SBELM_*im*_] in which SBELM_*ij*_ is trained by every pair of classes and its output is *p*_*ij*_(*t*_*i*_∣*β*, *x*) for sample *x* belonging to the *i*th against the *j*th class. The total number of classifiers is *m*(*m* − 1)/2.

In simultaneous failure diagnosis, more than one failure may occur at the same time that can infer the concept: ∑_*i*=1_^*m*^ *p*_*i*_ ≠ 1 [21]. By estimating each probability output of binary classifiers SBELM_*ij*_ to measure correlation between various classes, obtain the paired probability output *p*_*i*_ as follows:

(20) $$\begin{array}{c} p_{i}=\frac{\sum_{j=1,j\neq i}^{d}n_{ij}p_{ij}}{\sum_{j=1,j\neq i}^{d}n_{ij}},\;i=1,\ldots ,m, \end{array}$$

where *n*_*ij*_ is the number of training sample belonging to the *i*th and the *j*th class.

### 2.4. Optimization of Decision Threshold for Simultaneous Failure Mode Recognition

For a *m*-class classification problem, the output of classifiers based on PSBELM is a probability vector *P* = [*p*_1_,…, *p*_*m*_] in which *p*_*j*_ represents occurring possibility of the *j*th failure. In order to obtain final simultaneous failure modes, an appropriate threshold value is indispensable. In general, researchers usually use 0.5 to be the threshold value [21] which is of generality but not suitable for specific application. This paper utilizes Grid Search method and an independent sample set containing both single failure and simultaneous failure to generate an optimal decision threshold *ε* ^*^ between 0 and 1 which can convert probability output vector into result vector *F* = [*f*_1_,…, *f*_*i*_,…, *f*_*m*_] effectively:

(21) $$\begin{array}{c} f_{i}=\left\{\begin{array}{cc} 1 & p_{i}\geq \varepsilon^{*}\\ 0 & p_{i}<\varepsilon^{*}, \end{array}\right.\;i=1,\ldots ,m,\;\;\varepsilon^{*}\in \left(0,1\right). \end{array}$$

The simultaneous failure modes are those single failures that their corresponding *f*_*i*_ is equal to 1. Since the range of searching is limited, the time-consuming characteristic of Grid Search method can not weaken its advantages of global optimization compared with GA and PSO [18].

### 2.5. Evaluation of Performance Based on *F*_1_-Measure

Considering that partial matching is valid and significant in simultaneous failure diagnosis [22], utilize an independent testing set and *F*_1_-measure [23] which is commonly used for evaluation of information retrieval systems to evaluate diagnostic accuracy for the proposed simultaneous failure diagnostic framework. Given a data set *D* = (*x*_*i*_, *t*_*i*_), *i* = 1,…, *N*, *x*_*i*_ ∈ *R* ^*n*^, *t*_*i*_ ∈ *R* ^*m*^, *t*_*ij*_ ∈ {0,1}, *j* = 1,…, *m*, define two variables namely precision (*P*) and recall (*R*) among which *P* represents the ratio between correct identified single failure modes and the actual simultaneous failure modes and *R* represents the ratio between correct identified single failure modes and the predicted simultaneous failure modes:

(22) $$\begin{array}{c} P=\frac{\sum_{j=1}^{m}\sum_{i=1}^{N}f_{ij}^{*}t_{ij}}{\sum_{j=1}^{m}\sum_{i=1}^{N}t_{ij}},\;\;R=\frac{\sum_{j=1}^{m}\sum_{i=1}^{N}f_{ij}^{*}t_{ij}}{\sum_{j=1}^{m}\sum_{i=1}^{N}f_{ij}^{*}}, \end{array}$$

where *f*_i_^*^ = [*f*_*i*1_^*^,…, *f*_im_^*^] is the predicted simultaneous failure modes by using the proposed framework and *t*_*i*_ = [*t*_*i*1_,…, *t*_*im*_] is the actual simultaneous failure modes of *x*_*i*_. The *F*_1_-measure value can be obtained as follows:

(23) $$\begin{array}{c} E=\frac{2*P*R}{P+R}=\frac{2\sum_{j=1}^{m}\sum_{i=1}^{N}f_{ij}^{*}y_{ij}}{\sum_{j=1}^{m}\sum_{i=1}^{N}y_{ij}+\sum_{j=1}^{m}\sum_{i=1}^{N}f_{ij}^{*}}. \end{array}$$

### 2.6. The Proposed Framework for Final Drive Simultaneous Failure Diagnosis

**Figure 2:**
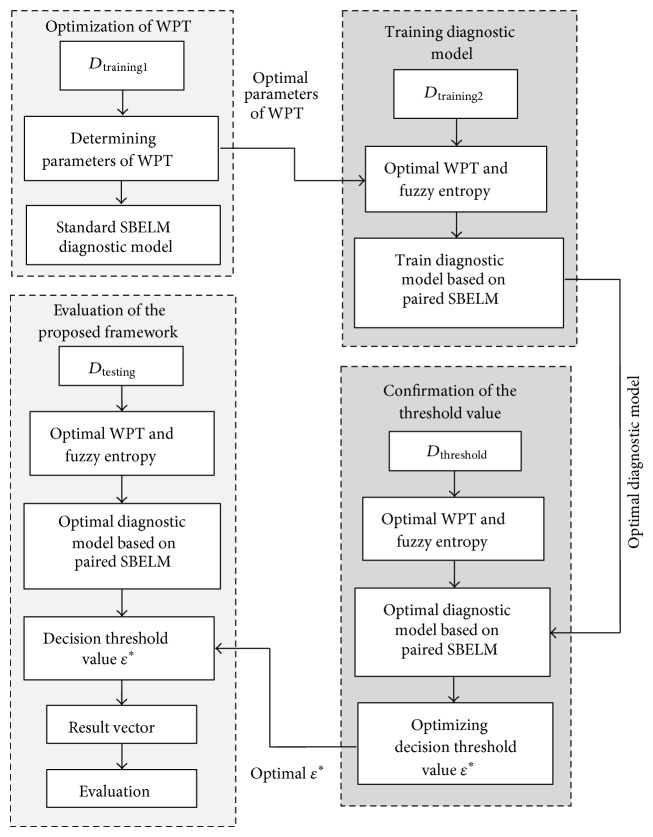
The structure of the proposed framework.

The structure of the proposed framework is shown in Figure 2 and the procedure of the proposed framework is described as follows.

1. Divide sample set into four parts: *D*_training1_, *D*_training2_, *D*_threshold_, and *D*_testing_. All the sample should be preprocessed by WPT and utilize fuzzy entropy to measure the feature of oscillatory.
2. Utilize *D*_training1_ containing only single failure modes to optimize parameters of WPT including number of layer *L* and mother wavelet in preprocessing by using failure diagnostic model of SBELM.
3. By using optimal parameters of WPT obtained from step 2, preprocess *D*_training2_ containing only single failure modes and train classifiers based on paired SBELM.
4. The optimum diagnostic model of PSBELM generates a probability output vector [*p*_1_,…, *p*_*m*_] in *m*-label classification problem and uses *D*_threshold_ containing both single and simultaneous failure modes and Grid Search to confirm the decision threshold value *ε* ^*^ which is used to obtain simultaneous failure modes.
5. Use *D*_testing_ and *F*_1_-measure to evaluate the diagnostic accuracy of the proposed framework.

## 3. Experiment Setup and Preprocessing

### 3.1. Experiment Setup

**Figure 3:**
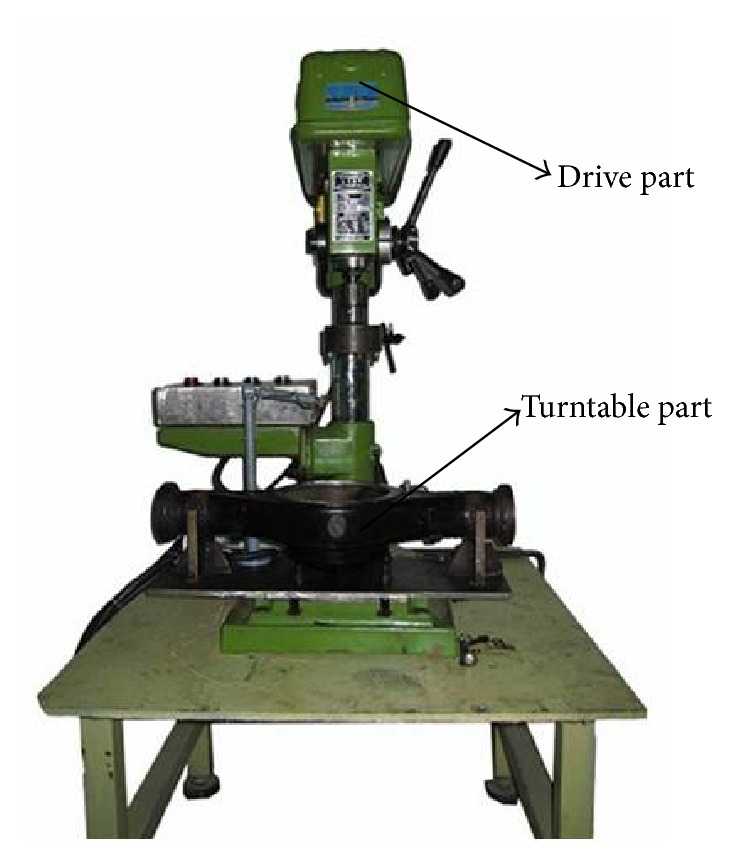
The test bed.

**Figure 4:**
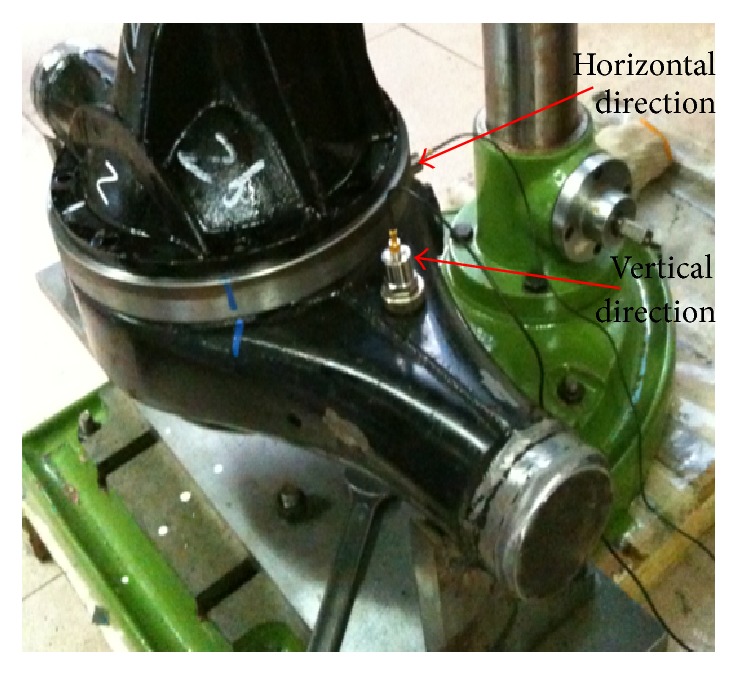
Two sensors on final drive.

**Figure 5:**
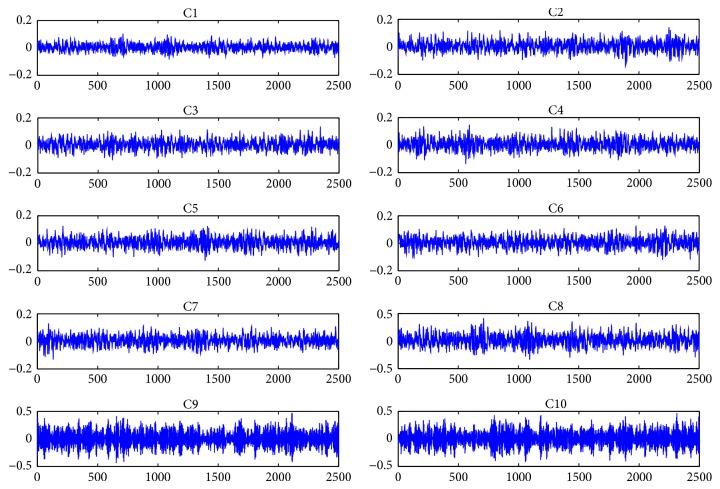
Vibration waveforms of 9 failure modes and normal status.

In order to obtain sample data with representativeness for constructing diagnostic model and verify the efficiency of the proposed diagnostic platform, implement the experiments on a test bed containing a PC, two sensors, a signal amplifier, and a simulation turntable with the composition as shown in Figure 3 in quiet room to collect enough original vibration signal of final drive. Two sensors are laid on the final drive in horizontal and vertical direction as shown in Figure 4 to collect vibration signal when it is set into running state. Most failures of final drive such as gear error, gear hard point, and tooth broken occurred in gear pair which is the hard core of final drive and consisted of a driving-gear and a driven-gear. In this research simulate 9 common failures including 6 single failures and 3 simultaneous failures which are described in detail in Table 1 under the rotating speed of 1200 r/m for driving motor. As shown in Figure 5, amplitudes of simultaneous failure are obviously greater than single failure because when simultaneous failure occurs these single failures are coupled together severely. The wave profiles between single and simultaneous failure patterns are similar, so that it is difficult to distinguish them manually but the characteristics embedded in each vibration signal can be extracted and identified by using methods afore mentioned.

Considering the universality of vibration signals which are used to construct diagnostic models, repeat simulating each failure mode for 100 times and record the most stable 2 seconds in each time with the sampling rate of 12 kHz which should be higher than the gear meshing frequency so that effective failure information may not be discarded during the sampling. Eventually, 1000 sample data are obtained and prepared to be preprocessed. All the simulations are implemented in MATLAB 7.0 which is running in a PC with CPU of 3.4 GHZ and RAM of 4.0 GB.

### 3.2. Feature Extraction Based on WPT and Fuzzy Entropy

In this paper, fuzzy entropy is used to reflect the change of complexity. By calculating the average fuzzy entropy of vibration signal corresponding to each failure mode which is shown in Table 2, we find out that the values of fuzzy entropy of these 10 failure modes are approximate so that it can not correctly distinguish failures of final drive. The reason is that information supplied by fuzzy entropy of original signal is limited and unable to reflect the deep-seated information of each failure situation. Therefore, we employ the frequently used mother wavelet function called Daubechies wavelet which has orthogonal characteristic and effectiveness in filtering signal of vibrating machinery to implement wavelet package transform and decompose original vibration signal into several subfrequency bands. By calculating fuzzy entropy of each frequency band obtained from *L*-level wavelet package transform decomposition, construct a feature vector with the dimension of 2^*L*^ which can effectively reflect the complexity and self-similarity of oscillation characteristic of failure modes occurring in final drive:

(24) $$\begin{array}{c} \text{Feature}=\left[\text{FuzzyEn}1,\text{FuzzyEn}2,\ldots ,\text{FuzzyEn}2^{L}\right]. \end{array}$$

### 3.3. Distribution Plan of Samples

There are 1000 sample data containing 100 normal samples, 600 single failure samples, and 300 simultaneous failure samples. In each trial, randomly divide the whole sample set into four parts: *D*_training1_, *D*_training2_, *D*_threshold_, and *D*_testing_. *D*_training1_ and *D*_training2_ which are consisting of only single failure modes are used for optimizing parameters of WPT and training optimal diagnostic model based on paired SBELM. *D*_threshold_ which contains both single and simultaneous failure modes is used to generate optimal decision threshold which convert probability result of diagnostic model based on paired SBELM into final simultaneous failure modes. *D*_testing_ is used to test and evaluate the proposed framework by using *F*_1_-measure. Ensure that the whole sample set be preprocessed and the size of training samples should be more than testing samples to ensure the generalization of the proposed framework. The distribution plan is shown in Table 3.

## 4. Result and Discussion

### 4.1. Optimization of Preprocessing and Feature Extraction

In data preprocessing, optimum combination of decomposition level and mother wavelet of WPT and parameters of fuzzy entropy can achieve better performance in classification. We use *D*_training1_ containing random 250 single samples to obtain the optimal combination of level number and mother wavelet which is suitable for preprocessing samples collected from final drive. In order to simplify experiment and on the basis of previous research result, focus on three wavelets Db3, Db4, and Db5 and three decomposition levels from 3 to 5. Three parameters of fuzzy entropy including *m*, *n*, and *r* are defined empirically in advance. Parameter *m* is usually set to be 2. Related to the boundary of fuzzy function, parameters *r* and *n* are setting as 0.2 and 2 STD where STD is the standard deviation of original data [8].

**Figure 6:**
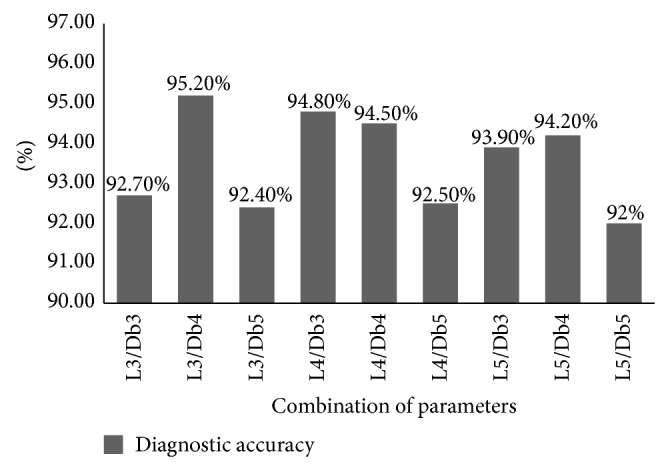
The diagnostic accuracy of different parameters.

By using single failure samples contained in *D*_training1_ and standard diagnostic model based on SBELM with failure parameters, find out appropriate parameters of WPT to achieve best performance of preprocessing. The standard diagnostic model based on SBELM is only used for selecting optimal parameters of WPT that exist in the best failure identification model in which the accuracy of classification is highest. The comparison result is shown in Figure 6 which indicates that classification accuracy of the preprocessing by using 3 level decomposition and Db4 as mother wavelet and standard diagnostic model based on SBELM is highest with the accuracy of 95.2%. This parameter combination of WPT is suitable for preprocessing the dataset in this application.

**Figure 7:**
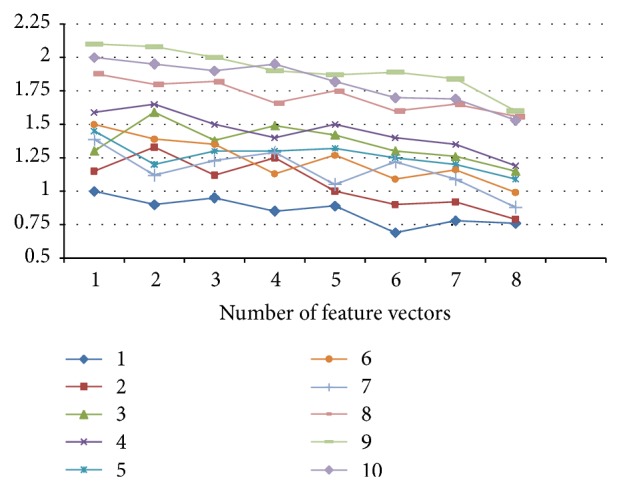
Mean value of fuzzy entropy for failure modes.

After decomposing vibration signal by using three-level wavelet package decomposition, calculate the corresponding value of fuzzy entropy as shown in Figure 7. In Figure 7, horizontal ordinate represents eight subfrequency bands of three-level wavelet package decomposition, and longitudinal coordinate represents the fuzzy entropy value. The FuzzyEn of the oscillation from final drive with simultaneous failures is larger than that of single failures and normal status. When simultaneous failures occur under rotation of gear pair, different failure points are coupling together to make the oscillation complex and stronger. Furthermore, the values of fuzzy entropy vary from one failure pattern to another. This characteristic denotes fuzzy entropy can be used as feature of failure diagnosis.

By calculating fuzzy entropy of each frequency band obtained from three-level wavelet package decomposition, we construct a feature vector with the dimension of 8 which can effectively reflect the failure modes of final drive:

(25) $$\begin{array}{c} \text{Feature}=\left[\text{FuzzyEn}1,\text{FuzzyEn}2,\ldots ,\text{FuzzyEn}8\right]. \end{array}$$

### 4.2. Effectiveness of Optimal Decision Threshold

After constructing optimal diagnostic model based on paired SBELM with optimal parameters of WPT in preprocessing by using only single failure modes, generation of optimal decision threshold is the pivotal point which affects final diagnostic accuracy of simultaneous failure. Traditional machine learning methods usually adopt 0.5 as general threshold value (GT) [24]. This research uses *D*_threshold_ containing 100 single failure modes and 200 simultaneous failure modes and Grid Search method with interval of 0.01 to search final decision threshold *ε* ^*^ in range of 0 to 1. Although Grid Search is time consuming, it can obtain global optimum.

**Figure 8:**
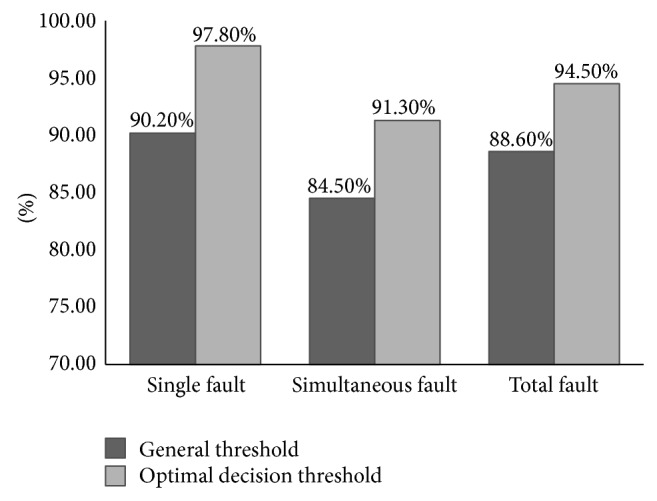
Diagnostic accuracies of models with general threshold and optimal decision threshold.

With the purpose of verifying effectiveness of optimal decision threshold, utilize 5-fold cross validation method to implement a set of experiments by using *D*_threshold_ for both single and simultaneous failure modes recognition. Results are shown in Figure 8.

After optimizing threshold, the accuracy of diagnostic model improves by an average of 6%. Fixed General threshold is generated by experience so that it has generalization but without optimization [25]. Even using the same diagnostic model to diagnose different sample set would require different threshold. Therefore, this research uses an independent sample set to generate optimal decision threshold.

### 4.3. Sensitivity Analysis of SBELM

**Figure 9:**
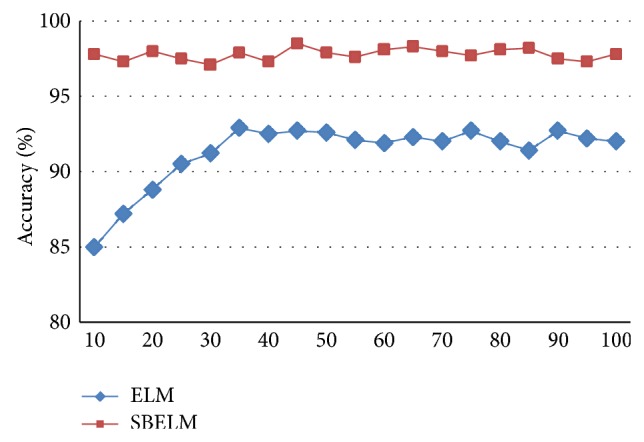
Variation of accuracy of ELM and SBELM.

For diagnosis based on ELM, diagnostic accuracy and training speed are sensitive to the initial number of hidden nodes. To analyze the sensitivity of SBELM on the number of hidden nodes in this application, use 500 single failure samples in *D*_training1_ and *D*_training2_ to train classifier based on SBELM and the best average accuracy along with the increase of hidden nodes is shown in Figure 9. As shown in Figure 9, the average accuracies of ELM with increment of hidden nodes are in larger variation. The reason for this fluctuation is that ELM is in poor generalization because of data overfitting [17]. However, the average accuracies are stable and are obviously higher than ELM. The result verifies that SBELM is relatively insensitive to the initial number of hidden nodes. Moreover, SBELM can obtain an excellent accuracy with a small hidden layer which reduces the computational cost effectively.

### 4.4. Evaluation of the Proposed Framework

In order to effectively confirm the availability of the proposed simultaneous failure diagnosis framework, we use *D*_testing_ containing 100 single failure modes and 100 simultaneous failure modes and *F*_1_-measure method to measure performance of the proposed framework and diagnostic model based on PNN and SVM in diagnostic accuracy and diagnostic speed. Firstly, use sample set which are consisting of *D*_training1_ and *D*_training2_ to construct and tune parameters of diagnostic model based on PNN and SVM separately and then use *D*_threshold_ to generate optimal threshold value. Since SVM is essentially used for binary-class classification [26], with the purpose of simultaneous failure diagnosis we combine SVM with multiclass classification strategy to construct a set of classifiers in which each classifier is only focusing on two failure modes. Trying to ensure the excellent performance of classifiers based on SVM, set the value of regularization parameter *C* of SVM to be 10^*α*^ where *α* is between 0 and 2. Radial basis kernel function is employed in SVM with *C* = 10 and *r* = 2 which show the best accuracy of classification. As a probability classifier, the crucial hyperparameter of PNN is spread *s*. In this research, the value of s is chosen from 1 to 3 with interval of 0.5 according to conclusion of references. Finally, the best hyperparameters *s* and threshold value *ε* for PNN are 1 and 0.69.

To verify the effectiveness of the paired strategy in the proposed framework, implement a set of experiments with one-to-all strategy. The experimental results are shown in Table 4. Comparing different classifiers with one-to-all strategy and paired strategy, the accuracies of classifiers with paired strategy are generally 2% to 4% higher than that of classifiers with one-to-all strategy. The primary reason is that paired strategy which is used in the proposed framework fully considers the correlation between each single failure. However, one-to-all strategy may cause some indecision regions between different classes. The indecision region is prone to sinking into misclassification.

To verify the performance of the proposed framework, implement a set of experiments about different classifiers with the same testing set and best parameters. The decision threshold values, training time, testing time, and testing accuracy of diagnostic models based on paired SBELM, SVM, and PNN are shown in Table 5. The diagnostic accuracy of paired SBELM for single failure, simultaneous failure, and entire sample is 98.4%, 92.8%, and 96.2% which are higher than that of the SVM and PNN. The reason is that SBELM estimates the probability distribution of output values instead of fitting data to improve generalization [17]. Moreover, the training time and testing time of paired SBELM are 145.4 ms and 48.7 ms that are much fewer than SVMs. The reason for this disparity is that even though paired SBELM builds a set of binary classifiers, the sparse characteristic of SBELM reduces the computational cost. Consequently, the disparity will become more obvious if the size of sample is big.

In practical application of auto manufacturer, representative and valid samples are continuously collected and added to the training sample database to improve training accuracy. Based on this, learning speed becomes a crucial factor for evaluating the efficiency of diagnostic platform. In general, considering both diagnostic accuracy and diagnostic efficiency, the proposed platform is superior in simultaneous failure diagnosis and it is not only suitable for final drive of car but also it can be porting to other research fields.

**Figure 10:**
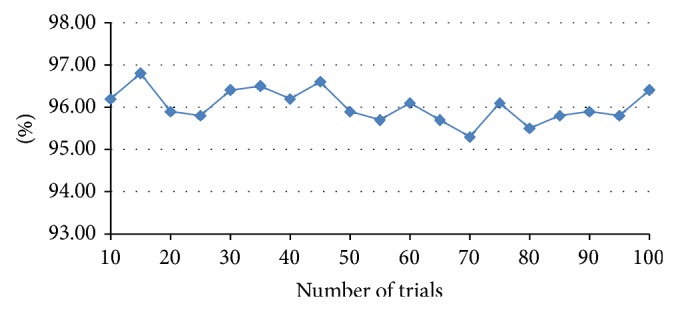
Testing result of 100 trials.

In order to verify the stability of the proposed diagnostic framework based on paired SBELM, implement 100 trials and in each trial the whole sample data is reshuffled and randomly distributed into *D*_testing_ afresh and make sure there are enough single failure samples and simultaneous failure samples in *D*_testing_. The testing result is shown in Figure 10 in which the testing accuracy is stable in the range between 95% and 97% and there is no dramatic variation in 100 simulation trials.

## 5. Conclusion

This paper proposes a novel framework based on SBELM and fuzzy entropy for simultaneous failure diagnosis of final drive which is hardcore to affect the performance and safety of car. The proposed framework contains four sections: preprocessing and feature extraction based on WPT and fuzzy entropy, construction of diagnostic model based on paired SBELM, generation of decision threshold value, and recognition of simultaneous failure modes. By using single failure samples, obtain optimal parameters of WPT which are perfectly adequate for the data in this application. Diagnostic model based on paired SBELM in which each binary classifier is trained by only single failure samples. With an independent sample subset containing both single and simultaneous failure samples, use Grid Search method to generate optimal decision threshold by which probability result obtained from diagnostic model can be converted into final result of simultaneous failure modes. Compared with frequently used diagnostic model based on SVM and PNN, there are three superiorities of the proposed framework. (1) The proposed framework based on SBELM inherits the advantages of ELM (efficient approximation and learning speed) and sparse Bayesian learning (high sparsity and generalization). (2) Fully considering the difficulty and impossibility of assembling all possible simultaneous failure modes, the proposed framework trains paired classifiers based on SBELM by using only single failure samples, and moreover the paired strategy can effectively avoid indecision regions between different classes which can result in misclassification. (3) With the average testing accuracy of 96.2% and testing time of 48.7 ms, the proposed framework outperforms other diagnostic models in diagnostic accuracy and learning speed. The proposed framework is general and transplantable for simultaneous failure diagnosis, so it can be applied to other applications in industrial area in which accuracy and time cost of failure identification are key factors.

## Figures and Tables

**Table 1:** Simple and simultaneous failure modes description.

Failure label		Failure mode description
C1	Single failures	Normal status

C2	Single failures	Gear error
C3		Gear burr
C4		Gear hard point
C5		Misalignment
C6		Gear tooth broken
C7		Gear crack

C8	Simultaneous failures	Gear tooth broken and gear crack
C9		Gear tooth broken and gear hard point
C10		Misalignment and gear crack

**Table 2:** Average fuzzy entropy of 10 failure modes.

	C1	C2	C3	C4	C5	C6	C7	C8	C9	C10
Average fuzzy entropy	1.7761	1.8695	1.8855	1.7904	1.8734	1.9018	1.8080	1.8653	1.8107	1.9268

**Table 3:** Division of the whole sample dataset.

	Single failure	Simultaneous failure	Total number
*D*_training1_	250		250
*D*_training2_	250		250
*D*_threshold_	100	200	300
*D*_testing_	100	100	200

Total	700	300	1000

**Table 4:** Comparison of paired strategy and one-to-all strategy.

Classifier	Decision threshold ∈[0,1]	Multiclass classification strategy	Accuracy (%)		
			Single failures	Simultaneous failures	Entire sample
PNN	0.69	One-to-all	91.54 (±1.02)	88.22 (±1.55)	89.42 (±1.67)
		paired strategy	93.21 (±1.25)	89.14 (±1.76)	92.33 (±2.05)

SVM	0.69	One-to-all	92.02 (±2.35)	80.90 (±1.62)	84.70 (±1.87)
		paired strategy	94.84 (±1.75)	84.32 (±1.35)	88.92 (±2.14)

SBELM	0.72	One-to-all	95.13 (±1.22)	90.54 (±2.05)	92.94 (±1.73)
		paired strategy	98.42 (±1.41)	92.81 (±2.35)	96.23 (±1.59)

**Table 5:** Performance of three classifiers.

	PNN	SVM	SBELM
Decision threshold [0,1]	0.69	0.69	0.76
Accuracy of single failure (%)	93.24 (±1.86)	94.81 (±2.19)	**98.42 (±1.55)**
Accuracy of simultaneous failure (%)	89.12 (±2.41)	87.34 (±1.96)	**92.81 (±1.85)**
Accuracy of entire sample (%)	92.30 (±2.55)	91.43 (±2.32)	**96.23 (±2.06)**
Training time (ms)	268.2	493	**145.4**
Testing time (ms)	86.5	119.4	**48.7**
